# Factors Associated with Not Breastfeeding and Delaying the Early Initiation of Breastfeeding in Mecca Region, Saudi Arabia

**DOI:** 10.3390/children5010008

**Published:** 2018-01-03

**Authors:** Firas S. Azzeh, Awfa Y. Alazzeh, Haifa H. Hijazi, Haneen Y. Wazzan, Monya T. Jawharji, Abdelelah S. Jazar, Amira M. Filimban, Ali S. Alshamrani, Mai S. Labani, Taghreed A. Hasanain, Ahmad A. Obeidat

**Affiliations:** 1 Department of Clinical Nutrition, Collage of Applied Medical Sciences, Umm Al-Qura University, Makkah 21955, Saudi Arabia; hofe_66@hotmail.com (H.H.H.); Hywazzan@uqu.edu.sa (H.Y.W.); mtjoharji@uqu.edu.sa (M.T.J.); abdelelahj@yahoo.com (A.S.J.); 2 Department of Clinical Nutrition, Faculty of Applied Medical Sciences, University of Ha’il, Ha’il 21955, Saudi Arabia; awfa.yosef@gmail.com; 3 Nutrition General Department, Awareness and Nutrition Education Division, Ministry of Health, Makkah 21955, Saudi Arabia; amfilimban@moh.gov.sa; 4 Pediatric Department, Maternity and Children Hospital, Makkah 21955, Saudi Arabia; ahadi@moh.gov.sa; 5 Medical Nutrition Therapy Department, Maternity and Children Hospital, Makkah 21955, Saudi Arabia; mlabani@moh.gov.sa (M.S.L.); Thasanain@moh.gov.sa (T.A.H.); 6 Department of Clinical Nutrition, Faculty of Applied Medical Sciences, Taibah University, Yanbu 21911, Saudi Arabia; aobeidat@taibahu.edu.sa

**Keywords:** breastfeeding, early initiation of breastfeeding, Baby-Friendly Hospital Initiative, Mecca

## Abstract

The objective of the study was to find the determinants related to not breastfeeding (BF) and others related to the delay in the early initiation of BF in the Mecca region, Saudi Arabia. A cross-sectional study in the Maternity and Children Hospital and primary healthcare centers was performed. A questionnaire was filled by dietitians to 814 asymptomatic Saudi mothers. Determinants related to not BF and the delay in the early initiation of BF were determined by binary logistic regression, and the odds ratio (OR) and 95% confidence interval (CI) were determined. Significant factors associated with not BF were not rooming-in infants in the mother’s room (OR: 2.37; 95% CI: 1.66–3.41) and using a pacifier (OR: 1.62; 95% CI: 1.13–2.33). The most significant determinant of the early initiation of BF was the initiation of bottle feeding (OR: 18.16; 95% CI: 10.51–31.4), followed by not rooming-in infants in the mother’s room (OR: 2.2; 95% CI: 1.52–3.18), initiation of partial feeding (OR: 1.89; 95% CI: 1.3–2.74), uninformed mothers regarding the importance of BF (OR: 1.56; 95% CI: 1.04–2.35), and cesarean sections (OR:1.42; 95% CI: 1.02–1.98). Risk factors affecting BF and the early initiation of BF in Mecca City should be highlighted in national campaigns to increase mothers’ awareness and promote BF practice.

## 1. Introduction

Breastfeeding (BF) provides multiple benefits for both newborns and mothers. Breast milk contains all the nutrients that a baby needs for the first 6 months of life [1]. The World Health Organization (WHO) [2] recommends mothers to start BF on the first hour after birth, which is referred as the “early initiation of BF”, and ensures that the infants have the colostrum, which is rich in protective factors for infants’ health. The initiation of BF within 1 h of birth has numerous immunological and nutritional benefits for newborns that have been found to reduce neonatal mortality [3]. A meta-analysis study reported that the early starting of BF was significantly linked with a reduction in all-cause neonatal mortality and low-birth-weight-related neonatal mortality among all live births [4]. Mechanisms have been proposed that would lead to a noticeable decrease in the mortality rate and these include early stimulation of the immune system through exposure to high levels of immunoglobulins and lymphocytes found in colostrum, along with the displacement of prelacteal feeds, which may be a vehicle for infectious pathogens and also disrupt normal gut maturation [4,5]. Furthermore, the early initiation of BF, alongside skin-to-skin contact, is recommended as one of several steps to prevent hypothermia in newborns [6].

Besides adopting the early initiation of BF, the international agencies recommend the exclusivity of BF for the child’s first 6 months and that BF continue for 24 months or beyond, which are suboptimal in most countries around the world [2]. In England, just over four out of five (83%) mothers started BF, but this high percentage in the initiation of BF has not been reflected to the same extent in its duration and exclusivity; by 6 weeks, the proportion of any BF dropped to 57%, and only 36% of mothers were still BF at 6 months [7]. A review in different Saudi Arabia cities found that the percentage of exclusive BF and the mean duration of BF were higher in Al-Hassa and Al-Taif regions and lower in Riyadh [8]. Moreover, the mean duration of BF in Saudi Arabia has showed a progressive decline since 1985 [8]. The WHO country profile for Saudi Arabia does not provide any data about BF types and durations or their determinants, because no recent or sufficient data are locally available [9]. Additionally, no current data have been found regarding the possible determinants of BF and the initiation of BF in Mecca City, besides only one investigation in 1992, which showed the risk factors associated with the duration of BF in one area of the Mecca region [10]. Therefore, the objective of the study is to provide updates about the possible risk factors related to not BF and other factors related to the delay in the early initiation of BF in the Mecca region of Saudi Arabia.

## 2. Materials and Methods

### 2.1. Subjects and Study Design

A cross-sectional descriptive study was conducted in the Mecca region, Saudi Arabia. Data were collected over 17 months, from June 2014 until November 2015. The participants were interviewed face-to-face in the Maternity and Children Hospital and primary healthcare centers located in Mecca City by trained dietitians. In this study, we included 814 randomly selected Saudi mothers, with an age range of 17–44 years, and having children aged 2 to 3 years old. The collected infants were 394 (48.4%) males and 420 (51.6%) females. We excluded mothers who delivered infants with any disability, genetic metabolic disorder, and/or congenital anomalies. A consent form was signed from all mothers before starting the interview. The ethical approval number, AMSEC-1-187-2014, following the rules of the Helsinki Declaration, was obtained from the Faculty of Applied Medical Sciences, Umm Al-Qura University.

### 2.2. Questionnaire

A closed pretested questionnaire consisting of three parts was used in this study. The questionnaire was designed and validated as discussed earlier in a previous paper by Azzeh [11]. The first part consisted of personal information, including the family size; the age, weight, height, and body mass index (BMI) of mothers; the family income; the mother’s occupation; and the parents’ educational level. The second part had questions related to the mother’s and child’s health status, such as the current birth, the type of delivery, the age of birth, the gender, the infant weight after birth, the mother’s diseases, the infant’s health issues after birth, the contraceptive method, and smoking. The third category of questions focused on postpartum conditions, which included the immediate place of the infant after birth, the initiation of feeding, the time of the first BF, the use of a pacifier, and whether mothers were informed about the importance of BF or not.

### 2.3. Statistical Analysis

Statistical analyses were done with SPSS software (Statistic Package for Social Sciences; IBM Corp., Armonk, NY, USA), version 20. The chi-square (*χ*^2^) test was used to find the association between categorical groups as a preliminary test for regression analysis. Binary logistic regression was performed to find the possible determinants of delayed early initiation of BF and not BF by the odds ratio (OR) and 95% confidence interval (CI) for different independent variables. To determine factors related to not BF, two groups were compared: mothers who did not breastfeed their children (*n* = 156) and mothers who breastfed their children for any type of BF (*n* = 658); to determine the possible factors associated with delaying the early initiation of BF, mothers who did not breastfeed their children (*n* = 156) were excluded from this analysis. Therefore, mothers who initiated BF early (≤1 h; *n* = 310) were compared with mothers who delayed BF (>1 h; *n* = 348) in the logistic regression. A *p*-value of <0.05 was considered statistically significant.

## 3. Results

The sample characteristics are shown in Table 1. The average age (±standard deviation (SD)) was 29.8 ± 6.2 years, and 269 mothers (33%) were in the age category of 25–29 years. The average BMI was 25.5 ± 5.3 kg/m^2^, and 399 mothers (49%) were in the normal weight category. The household size ranged from 3–13 members with an average value of 4.8 ± 1.7, and 69.9% of their family size were ≤5 members. The medium income category was the majority: 47.3% at around 5000–10,000 Saudi Riyal (SR). Five hundred and eighty-four (71.7%) mothers and 583 (71.6%) fathers had an academic degree. The majority of the surveyed mothers were unemployed (*n* = 563; 69.2%). Overall, 156 mothers (19.2%) did not breastfeed their children for any duration, the early initiation of BF (≤1 h) was observed in 310 (38.1%) mothers, and 348 (42.7%) mothers delayed the initiation of BF for >1 h. Table 1 also shows the *χ*^2^ values and binary logistic regression for sociodemographic characteristics as predictors for not BF. Family size, age categories, BMI categories, income level, the mother’s education, the father’s education, and the mother’s working showed no effects on BF.

The study results demonstrated that health-related characteristics of mothers and infants had no contribution to BF or not (Table 2). These factors were the current birth, the type of the delivery, the infant’s gender, the age of birth, the infant’s birth weight, the mother or child suffering from any disease, contraceptive usage, mother’s smoking, and second-hand smoking.

Table 3 determines the BF habits and patterns as predictors for not BF. Mothers who used a pacifier during the first 6 months of the infant’s life showed an OR of 1.62 (95% CI: 1.13–2.33) compared to mothers who did not use a pacifier. Not rooming-in infants in the mother’s room after delivery showed 2.37 higher odds (95% CI: 1.66–3.41) than babies placed in the mother’s room after delivery. The time of initiation of BF and whether mothers were informed about the importance of BF did not affect BF (Table 3).

Table 4, Table 5 and Table 6 represent the sociodemographic characteristics, health-related characteristics of mothers and infants, and BF habits and patterns as predictors for delaying the early initiation of BF, respectively. There were no significant relations between sociodemographics and delaying the early initiation of BF. The Table 5 results show that a caesarean section was the only significant (*p* < 0.05) predictor for delaying BF by >1 h, and the OR was 1.42 (95% CI: 1.02–1.98). Data in Table 6 shows that the placement of the baby in a separate room immediately after birth (OR: 2.2; 95% CI: 1.52–3.18), partial (OR: 1.89; 95% CI: 1.3–2.74) or bottle (OR: 18.16; 95% CI: 10.51–31.4) initiation of feeding, and mothers who did not receive information about the importance of BF (OR: 1.56; 95% CI: 1.04–2.35) were the significant (*p* < 0.05) factors associated with delaying BF by >1 h.

## 4. Discussion

Our study examined the possible risk factors related to not BF and other factors associated with delaying the early initiation of BF (>1 h) in the Mecca region of Saudi Arabia. The study results showed that 19.2% of mothers were not BF and 42.7% delayed the initiation of BF by >1 h. Our key findings for factors associated with not BF were placing the baby in a separate room after delivery and using a pacifier. On the other hand, factors related to the delay in the early initiation of BF were a cesarean section, not rooming-in infants in the mother’s room, early use of formula milk, and uninformed mothers about the importance of BF.

The most common factor associated with both not BF and a delay in the early initiation of BF was not rooming-in infants in the mother’s room after delivery. Rooming-in infants in the mother’s room is not mandatory in the Maternity and Children Hospital in Mecca because it is not designated as a baby-friendly hospital through the Baby-Friendly Hospital Initiative (BFHI). The BFHI has been developed since 1993 by the WHO and the United Nations Children’s Fund (UNICEF) [12] to encourage BF practice, and to provide practices to promote, protect and support women’s BF by implementing 10 steps in hospitals. These steps are (i) having a written BF policy that is regularly communicated to all healthcare professionals; (ii) all healthcare providers being trained to implement this policy; (iii) all mothers being informed about the benefits of BF; (iv) mothers initiating BF within a half-hour after childbirth; (v) mothers being trained for BF and sustaining BF; (vi) no food, water, drink, or formula milk being given to newborn infants unless medically required; (vii) rooming-in infants in the mother’s room 24 h a day; (viii) promoting BF on demand; (ix) no artificial teats or pacifiers for infants; and finally (x) encouraging the grouping of mothers for BF after being discharged from the hospital. The initiative is applied in more than 152 countries worldwide, with reports of increased exclusive BF and the early initiation of BF following its establishment [13]. However, only 28 hospitals in Saudi Arabia have been monitored as BFHI hospitals until 2016 [14]. A study among 31 maternity hospitals in Belarus showed that mothers inside BFHI institutes tended to have longer BF periods than mothers who did not [15]. The present study results found that infants who were placed in a separate room after birth were about 2.37-fold not breastfed and that 2.2-fold had delayed BF by >1 h when compared to infants roomed-in in the mother’s room immediately after birth, and the absence of rooming-in will likely affect exclusive BF. A study in the United Arab Emirates reported that mothers who kept their infants in the same room after delivery had a rate of BF 6 times higher than mothers who kept their infants in separate rooms [16]. Approximately 41.7% of mothers who used a pacifier for their infants had odds 1.6 times higher for not BF relative to mothers who did not use the pacifier. In addition, mothers who partially initiated BF showed a 1.89 times delayed BF (>1 h) compared to mothers who initiated BF exclusively. Moreover, uninformed mothers about the benefits of BF had 1.56 times higher odds of delaying the early initiation of BF (>1 h) as compared to informed mothers. Therefore, this study accentuates the importance of adopting the BFHI’s policies in Mecca hospitals to promote BF practices.

In the present study, the use of pacifiers was significantly associated with not BF. Some studies have revealed a negative association between pacifier use and BF duration [17,18], even in Mecca City [11]. Those studies observed that pacifier use would lead to early weaning, as the use of pacifiers would decrease BF motivation and therefore difficulties would arise. However, other studies have showed a positive effect of pacifier use by diminishing the risk of sudden infant death syndrome [19,20]. The American Academy of Pediatrics encourages the use of pacifiers after BF at naptime and bedtime for infants over 1 month old. This piece of information was used by the American Academy of Pediatrics Task Force to suggest that the use of pacifiers after the first month does not increase the risk of BF cessation [21]. BF and pacifier use are influenced by several cultural and psychological factors that are likely not easy to measure in observational studies [22]. Therefore, longitudinal investigations are recommended to find clear results and associations between pacifier use and BF duration.

Some major risks for delaying BF initiation were caesarean delivery, the use of anesthesia, tiredness of the mother and insufficient maternal skills to initiate BF [23,24,25], as well as the increased risk of prelacteal feeding [3]. The study results found that mothers who had a caesarean delivery had about 1.42 times higher odds for delaying BF by >1 h when compared to mothers who had a vaginal delivery. Our results were consistent with those found by Batal et al. [26] and Radwan [16], who reported that hospital-related factors and cesarean sections significantly resulted in delays in BF initiation.

The religious and cultural environment in Saudi Arabia is supportive of BF [27]. However, the BF rate has decreased in recent years, likely as a result of changes in lifestyle habits, mothers working, and other factors [11]. In the present study, the early initiation of BF was significantly affected by thhe partial initiation of BF or the early introduction of bottle feeding. The rate of formula feeding in Saudi Arabia was higher than the global average rate [14]. A national survey conducted by the International Baby Food Action Network showed that milk formula use in Saudi Arabia was 51%, 76%, and 90% at 1, 3, and 6 months of infants’ age, respectively, compared with the 31% average rate from 33 countries [14]. A study in Mecca found that 86% of mothers supplemented their infants with milk formula during the first 6 months, and the median infant’s age of starting milk formula was 3 months [11]. However, Mosher et al. [14] found that milk formula use in BFHI hospitals was less than for non-BFHI hospitals. This is likely as a result of educational impact by BFHI hospitals. In addition, mixed feeding rather than exclusive formula feeding was more likely to be practiced by women in the BFHI hospital when formula feed was introduced, and other women tended to switch from mixed feeding to exclusive BF [28]. In Saudi Arabia, the optional adoption of the BFHI may hinder its implementation by hospitals. Until this time, no legislation has been adopted to enhance BF practices for Saudi mothers. This critical issue should be a high priority of the Ministry of Health to encourage sustaining BF for 24 months, even in workplaces, as well as to establish well-designed facilities in the Saudi community to promote BF at any time according to infants’ demand. The United Arab Emirates is the leading country in the Mediterranean region in this aspect, with a successful implementation of the BFHI in all maternity hospitals [29]. Some challenges may arise during practicing BFHI steps, such as initial resistance by hospital staff, the need to change current common practices from health professionals, the long time that is required for BF data collection and maternal education, BF not being seen as a high priority by some mothers, and the high budget needed for implementing BFHI steps [29].

The city of Mecca has multiracial citizens, a possible confounding factor that was not measured in this study. This investigation was also limited by its design as a retrospective cross-sectional study.

## 5. Conclusions

The study results showed that placing babies in a separate room after delivery and using a pacifier are factors associated with BF outcomes in Saudi Arabia. On the other hand, the initiation of the first BF within an hour is affected by cesarean sections, not rooming-in infants in the mother’s room, the early use of formula milk, and uninformed mothers about the importance of BF. BFHI policies should be adopted as soon as possible in the Maternity and Children Hospital in Mecca to enhance both the early initiation of BF and BF duration. Additionally, national campaigns about possible determinants of BF and the early initiation of BF should be developed for Saudi mothers.

## Figures and Tables

**Table 1:** Sociodemographic characteristics as predictors for not breastfeeding (BF).

Parameter	Frequency (%) or Mean ± SD			*p*-Value (*χ*^2^)	OR (95% CI)
	Total (*N* = 814)	BF			
		Yes	No		
		(*n* = 658)	(*n* = 156)		
Family size	4.8 ± 1.7				
≤5	569 (69.9)	450 (68.4)	119 (76.3)	0.032	1.49 (0.99–2.23)
>6	245 (30.1)	208 (31.6)	37 (23.7)	(3.73)	1
Age category (years)	29.8 ± 6.2				
≤19	14 (1.7)	11 (1.7)	3 (1.9)	0.937	1.24 (0.37–4.711)
20–24	167 (20.5)	136 (20.7)	31 (19.9)	(0.81)	1.04 (0.6–1.8)
25–29	269 (33)	213 (32.4)	56 (35.9)		1.2 (0.73–1.95)
30–34	192 (23.6)	157 (23.9)	35 (22.4)		1.01 (0.59–1.73)
≥35	172 (21.1)	141 (21.4)	31 (19.9)		1
BMI category (kg/m^2^)	25.5 ± 5.3				
Underweight	30 (3.7)	26 (4)	4 (2.6)	0.781	0.65 (0.22–1.93)
Normal	399 (49)	323 (49.1)	76 (48.7)	(1.08)	1
Overweight	244 (30)	198 (30.1)	46 (29.5)		0.99 (0.66–1.48)
Obese	141 (17.3)	111 (16.9)	30 (19.2)		1.15 (0.715–1.85)
Income level (SR)					
<5000	142 (17.4)	117 (17.8)	25 (16)	0.475	0.87 (0.41–1.88)
5000–10,000	385 (47.3)	303 (46)	82 (52.6)	(2.5)	1.11 (0.56–2.17)
10,000–20,000	226 (27.8)	189 (28.7)	37 (23.7)		0.8 (0.39–1.65)
>20,000	61 (7.5)	49 (7.4)	12 (7.7)		1
Mother’s education					
Illiterate	12 (1.5)	9 (1.4)	3 (1.9)	0.574	1.52 (0.41–5.713)
Read and write	34 (4.2)	27 (4.1)	7 (4.5)	(1.99)	1.18 (0.5–2.79)
Intermediate	184 (22.6)	143 (21.7)	41 (26.3)		1.31 (0.87–1.96)
Academic	584 (71.7)	479 (72.8)	105 (67.3)		1
Father’s education					
Illiterate	6 (0.7)	4 (0.6)	2 (1.3)	0.752	2.15 (0.39–11.89)
Read and write	32 (3.9)	27 (4.1)	5 (3.2)	(1.21)	0.8 (0.3–2.11)
Intermediate	193 (23.7)	154 (23.4)	39 (25)		1.1 (0.72–1.64)
Academic	583 (71.6)	473 (71.9)	110 (70.5)		1
Mother works					
Yes	251 (30.8)	207 (31.5)	44 (28.2)	0.245	0.43 (0.58–1.26)
No	563 (69.2)	451 (68.5)	112 (71.8)	(0.626)	1

Dependent variable: any type of breastfeeding. Abbreviations: CI: confidence interval; OR: odds ratio; SD: standard deviation, BMI: body mass index, SR: Saudi Riyal; *χ*^2^: chi-square.

**Table 2:** Health-related characteristics of mothers and infants as predictors for not BF.

Parameter	Frequency (%) or Mean ± SD			*p*-Value (*χ*^2^)	OR (95% CI)
	Total (*N* = 814)	BF			
		Yes	No		
		(*n* = 658)	(*n* = 156)		
Current birth					
First	279 (34.3)	213 (32.4)	66 (42.3)	0.123	1.52 (0.97–2.4)
Second	201 (24.7)	169 (25.7)	32 (20.5)	(5.78)	0.93 (0.55–1.57)
Third	121 (14.9)	99 (15)	22 (14.1)		1.09 (0.61–1.96)
Fourth or more	213 (26.2)	177 (26.9)	36 (23.1)		1
Type of delivery					
Normal	558 (68.6)	452 (68.7)	106 (67.9)	0.464	1
Cesarean	256 (31.4)	206 (31.3)	50 (32.1)	(0.032)	1.04 (0.71–1.51)
Gender					
Male	394 (48.4)	317 (48.2)	77 (49.4)	0.43	1.05 (0.74–1.49)
Female	420 (51.6)	341 (51.8)	79 (50.6)	(0.071)	1
Age of birth					
9th month	718 (88.2)	583 (88.6)	135 (86.5)	0.769	1
≤8th month	42 (5.2)	33 (5)	9 (5.8)	(0.53)	1.18 (0.55–2.52)
≥10th month	54 (6.6)	42 (6.4)	12 (7.7)		1.23 (0.63–2.41)
Childbirth weight (kg)					
≤2.5	225 (27.6)	171 (26)	54 (34.6)	0.165	1.64 (1.01–2.67)
2.6–3	335 (41.2)	274 (41.6)	61 (39.1)	(5.09)	1.16 (0.72–1.85)
3.1–3.5	198 (24.3)	166 (25.2)	32 (20.5)		1
≥3.6	56 (6.9)	47 (7.1)	9 (5.8)		0.99 (0.44–2.23)
Mother suffers from chronic disease(s)					
Yes	98 (12)	83 (12.6)	15 (9.6)	0.186	0.74 (0.41–1.32)
No	716 (88)	575 (87.4)	141 (90.4)	(1.07)	1
Child suffers from postnatal disease(s)					
Yes	64 (7.9)	48 (7.3)	16 (10.3)	0.143	1.45 (0.8–2.63)
No	750 (92.1)	610 (92.7)	140 (89.7)	(1.53)	1
Contraceptive use					
No use	248 (30.5)	190 (28.9)	58 (37.2)	0.118	1.52 (1.01–2.28)
Hormonal	226 (27.8)	185 (28.1)	41 (26.3)	(4.27)	0.67 (0.71–1.71)
Not hormonal	340 (41.8)	283 (43)	57 (36.5)		1
Mother smokes					
Yes	75 (9.2)	59 (9)	16 (10.3)	0.356	1.16 (0.65–2.08)
No	739 (90.8)	599 (91)	140 (89.7)	(0.25)	1
Second-hand smoker					
Yes	320 (39.3)	256 (38.9)	64 (41)	0.345	1.09 (0.77–1.56)
No	494 (60.7)	402 (61.1)	92 (59)	(0.24)	1

Dependent variable: any type of breastfeeding.

**Table 3:** Breastfeeding habits and patterns as predictors for not BF.

Parameter	Frequency (%) or Mean ± SD			*p*-Value (*χ*^2^)	OR (95% CI)
	Total (*N* = 814)	BF			
		Yes	No		
		(*n* = 658)	(*n* = 156)		
Rooming-in in mother’s room					
Yes	576 (70.8)	490 (74.5)	86 (55.1)	<0.001	1
No	238 (29.2)	168 (25.5)	70 (44.9)	(22.8)	2.37 (1.66–3.41) ***
Initiation of BF (*n* = 658)					
≤1 h	310 (47.1)	309 (47.2)	1 (25)	0.357	1
>1 h	348 (52.9)	345 (52.8)	3 (75)	(0.79)	2.69 (0.28–26)
Using a pacifier for infants					
Yes	266 (32.7)	201 (30.5)	65 (41.7)	0.006	1.62 (1.13–2.33) **
No	548 (67.3)	457 (69.5)	91 (58.3)	(7.09)	1
Mother informed on the importance of BF					
Yes	665 (81.7)	539 (81.9)	126 (80.8)	0.408	1
No	149 (18.3)	119 (18.1)	30 (19.2)	(0.11)	1.08 (0.69–1.68)

Dependent variable: any type of breastfeeding. ** significant at *p* < 0.01; *** significant at *p* < 0.001.

**Table 4:** Sociodemographic characteristics as predictors for delaying BF by >1 h.

Parameter	Frequency (%) or Mean ± SD			*p*-Value (*χ*^2^)	OR (95% CI)
	Total (*N* = 658)	Early BF			
		Yes	No		
		(*n* = 310)	(*n* = 348)		
Family size					
≤5	448 (68.1)	211 (68.1)	237 (68.1)	0.529	1 (0.72–1.39)
>6	210 (31.9)	99 (31.9)	111 (31.9)	(0)	1
Age category (years)					
≤19	11 (1.7)	3 (1)	8 (2.3)	0.489	2.04 (0.52–8.01)
20–24	136 (20.7)	67 (21.6)	69 (19.8)	(3.43)	0.79 (0.49–1.26)
25–29	213 (32.4)	106 (34.2)	107 (30.7)		0.77 (0.51–1.18)
30–34	155 (23.6)	72 (23.2)	83 (23.9)		0.88 (0.56–1.39)
≥35	143 (21.7)	62 (20)	81 (23.3)		1
BMI category (kg/m^2^)					
Underweight	26 (4)	11 (3.5)	15 (4.3)	0.379	1.17 (0.52–2.62)
Normal	321 (48.8)	148 (47.7)	173 (49.7)	(3.08)	1
Overweight	201 (30.5)	91 (29.4)	110 (31.6)		1.03 (0.73–1.47)
Obese	110 (16.7)	60 (19.4)	50 (14.4)		0.71 (0.46–1.1)
Income level (SR)					
<5000	117 (17.8)	56 (18.1)	61 (17.5)	0.705	0.856 (0.44–1.67)
5000–10,000	303 (46)	149 (48.1)	154 (44.3)	(1.4)	0.81 (0.45–1.48)
10,000–20,000	188 (28.6)	83 (26.8)	105 (30.2)		0.99 (0.53–1.86)
>20,000	50 (7.6)	22 (7.1)	28 (8)		1
Mother’s education					
Illiterate	9 (1.4)	5 (1.6)	4 (1.1)	0.576	0.68 (0.18–2.57)
Read and write	27 (4.1)	11 (3.5)	16 (4.6)	(1.98)	1.24 (0.56–2.73)
Intermediate	144 (21.9)	74 (23.9)	70 (20.1)		0.81 (0.56–1.17)
Academic	478 (72.6)	220 (71)	258 (74.1)		1
Father’s education					
Illiterate	4 (0.6)	3 (1)	1 (0.3)	0.479	0.3 (0.03–2.89)
Read and write	27 (4.1)	15 (4.8)	12 (3.4)	(2.48)	0.72 (0.33–1.57)
Intermediate	151 (22.9)	67 (21.6)	84 (24.1)		1.12 (0.18–1.62)
Academic	476 (72.3)	225 (72.6)	251 (72.1)		1
Mother works					
Yes	210 (31.9)	98 (31.6)	112 (32.2)	0.471	1.03 (0.74–1.43)
No	448 (68.1)	212 (68.4)	236 (67.8)	(0.03)	1

Dependent variable: <1 h.

**Table 5:** Health-related characteristics of mothers and infants as predictors for delaying BF by >1 h.

Parameter	Frequency (%) or Mean ± SD			*p*-Value (*χ*^2^)	OR (95% CI)
	Total (*N* = 658)	Early BF			
		Yes	No		
		(*n* = 310)	(*n* = 348)		
Current birth					
First	213 (32.4)	98 (31.6)	115 (33)	0.854	1.16 (0.78–1.73)
Second	170 (25.8)	80 (25.8)	90 (25.9)	(0.78)	1.11 (0.73–1.7)
Third	98 (14.9)	44 (14.2)	54 (15.5)		1.21 (0.74–1.99)
Fourth or more	177 (26.9)	88 (28.4)	89 (25.6)		1
Type of delivery					
Normal	449 (68.2)	224 (72.3)	225 (64.7)	0.044	1
Cesarean	209 (31.8)	86 (27.7)	123 (35.3)	(3.18)	1.42 (1.02–1.98) *
Gender					
Male	317 (48.2)	150 (48.4)	167 (48)	0.49	0.98 (0.72–1.34)
Female	341 (51.8)	160 (51.6)	181 (52)	(0.01)	1
Age of birth					
9th month	582 (88.4)	281 (90.6)	301 (86.5)	0.246	1
≤8th month	33 (5)	13 (4.2)	20 (5.7)	(2.8)	1.44 (0.7–2.94)
≥10th month	43 (6.5)	16 (5.2)	27 (7.8)		1.58 (0.83–3)
Baby’s weight at birth (kg)					
≤2.5	171 (26)	82 (26.5)	89 (25.6)	0.47	0.95 (0.62–1.46)
2.6–3	275 (41.8)	124 (40)	151 (43.4)	(2.53)	1.07 (0.72–1.57)
3.1–3.5	165 (25.1)	77 (24.8)	88 (25.3)		1
≥3.6	47 (7.1)	27 (8.7)	20 (5.7)		0.65 (0.34–1.25)
Mother suffers from chronic disease(s)					
Yes	83 (12.6)	35 (11.3)	48 (13.8)	0.199	1.26 (0.79–2)
No	252 (87.4)	275 (88.7)	300 (86.2)	(0.93)	1
Child suffers from postnatal disease(s)					
Yes	48 (7.3)	26 (7.5)	22 (7.1)	0.487	1.06 (0.59–1.91)
No	610 (92.7)	322 (92.5)	288 (92.9)	(0.03)	1
Contraceptive use					
No use	192 (29.2)	93 (30)	99 (28.4)	0.751	0.89 (0.61–1.28)
Hormonal	182 (27.7)	88 (28.4)	94 (27)	(0.753)	0.89 (0.61–1.29)
Not hormonal	284 (43.2)	129 (41.6)	155 (44.5)		1
Mother smokes					
Yes	57 (8.7)	25 (8.1)	32 (9.2)	0.354	1.15 (0.69–2)
No	601 (91.3)	285 (91.9)	316 (90.8)	(0.27)	1
Second-hand smoker					
Yes	254 (38.9)	125 (40.3)	131 (37.6)	0.266	0.89 (0.65–1.22)
No	402 (61.1)	185 (59.7)	217 (62.4)	(0.5)	1

Dependent variable: <1 h. * Significant at *p* < 0.05.

**Table 6:** Breastfeeding (BF) habits and patterns as predictors for delaying BF by >1 h.

Parameter	Frequency (%) or Mean ± SD			*p*-Value (*χ*^2^)	OR (95% CI)
	Total (*N* = 658)	Early BF			
		Yes	No		
		(*n* = 310)	(*n* = 348)		
Rooming-in in mother’s room					
Yes	491 (74.6)	255 (82.3)	236 (67.8)	<0.001	1
No	167 (25.4)	55 (17.7)	112 (32.2)	(18.1)	2.2 (1.52–3.18) ***
Initiation of feeding					
Exclusive BF	280 (42.6)	188 (60.6)	92 (26.4)	<0.001	1
Partial BF	200 (30.4)	104 (33.5)	96 (27.6)	(144.8)	1.89 (1.3–2.74) **
Exclusive formula feeding	178 (27.1)	18 (5.8)	160 (46)		18.16 (10.51–31.4) ***
Using a pacifier for infants					
Yes	203 (30.9)	96 (31)	107 (30.7)	0.509	0.99 (0.71–1.38)
No	455 (69.1)	214 (69)	241 (69.3)	(0)	1
Mother informed on the importance of BF					
Yes	540 (82.1)	265 (85.5)	275 (79)	0.02	1
No	118 (17.9)	45 (15.4)	73 (21)	(4.65)	1.56 (1.04–2.35) *

Dependent variable: <1 h. * Significant at *p* < 0.05; ** significant at *p* < 0.01; *** significant at *p* < 0.001.
